# A Three-Monoclonal Antibody Combination Potently Neutralizes BoNT/G Toxin in Mice

**DOI:** 10.3390/toxins15050316

**Published:** 2023-04-30

**Authors:** Yongfeng Fan, Jianlong Lou, Christina C. Tam, Weihua Wen, Fraser Conrad, Priscila Leal da Silva Alves, Luisa W. Cheng, Consuelo Garcia-Rodriguez, Shauna Farr-Jones, James D. Marks

**Affiliations:** 1 Department of Anesthesia and Perioperative Care, University of California, 1001 Potrero Ave., San Francisco, CA 94110, USA; 2 Western Regional Research Center, Agricultural Research Station, United States Department of Agriculture, Albany, CA 94710, USA

**Keywords:** botulinum neurotoxin, oligoclonal antibodies, serotype G botulism, recombinant antibodies, antibody engineering, mouse neutralization assay

## Abstract

Equine-derived antitoxin (BAT^®^) is the only treatment for botulism from botulinum neurotoxin serotype G (BoNT/G). BAT^®^ is a foreign protein with potentially severe adverse effects and is not renewable. To develop a safe, more potent, and renewable antitoxin, humanized monoclonal antibodies (mAbs) were generated. Yeast displayed single chain Fv (scFv) libraries were prepared from mice immunized with BoNT/G and BoNT/G domains and screened with BoNT/G using fluorescence-activated cell sorting (FACS). Fourteen scFv-binding BoNT/G were isolated with K_D_ values ranging from 3.86 nM to 103 nM (median K_D_ 20.9 nM). Five mAb-binding non-overlapping epitopes were humanized and affinity matured to create antibodies hu6G6.2, hu6G7.2, hu6G9.1, hu6G10, and hu6G11.2, with IgG K_D_ values ranging from 51 pM to 8 pM. Three IgG combinations completely protected mice challenged with 10,000 LD_50_s of BoNT/G at a total mAb dose of 6.25 μg per mouse. The mAb combinations have the potential for use in the diagnosis and treatment of botulism due to serotype G and, along with antibody combinations to BoNT/A, B, C, D, E, and F, provide the basis for a fully recombinant heptavalent botulinum antitoxin to replace the legacy equine product.

## 1. Introduction

Botulinum neurotoxin (BoNT) is the most toxic substance known, with the estimated human LD_50_ for inhalation being 1 to 3 nanograms of toxin/kilogram body mass [1]. Seven serotypes of BoNT/A-G are well documented, with BoNT/H (or HA) [2,3] being recently reported, as well as other BoNT-like proteins: BoNT/X [4], eBoNT/J [5] and BoNT/Wo [6].

Most naturally occurring human botulism is caused by BoNT serotypes A, B, E, and F and is only rarely associated with BoNT/G. BoNT/G was suspected as the cause of death in four adults and an infant in Switzerland [7], confirming the human lethality of this serotype in humans. An open bone fracture resulting in wound botulism was suspected to be associated with BoNT/G since the 16 s rRNA gene of *C. botulinum* type G (now *C. argentinense* [8]) was identified in the operative cultures [9]. BoNT/G-producing clostridia species can be found in soil in Switzerland [10]. Given the potential of BoNT/G to cause human intoxication, the US government requires a heptavalent antitoxin that includes BoNT/G for stockpiling.

BoNT/G is produced by *C. argentinense* as a single-chain protein of around 150 kDa [11,12]. No subtypes of BoNT/G have been reported, unlike serotypes A, B, E, and F. With trypsin digestion, BoNT/G releases a light chain (LC) that is a catalytic zinc-dependent metalloprotease (~50 kDa) and a heavy chain (HC) that is composed of a translocation domain (H_N_, 50 kDa) and a receptor-binding domain (H_C_, 50 kDa). BoNT/G binds to all the polysialogangliosides on the neuronal membrane and synaptic vessel protein 2 (SV2) [13]. The crystal structure of the BoNT/G binding domain has been determined [14]. The HC and LC are connected by a disulfide bond between LC and H_N_ [15]. BoNT/G shares the most similarity to the BoNT/B subtype Okra (58.2%), whereas the similarity to other serotypes ranges from 32.8 to 38%, with the least similarity to the BoNT/C Stockholm subtype [11,16]. The BoNT binding domain binds synaptotagmins I and II on neurons [17,18,19], while the translocation domain forms a channel through which the catalytic domain (LC) is delivered into neurons. In the neurons, the catalytic domain cleaves one of the SNARE proteins, synaptobrevin-2, at Ala80–Ala81 [15] and therefore blocks the release of the neurotransmitter acetylcholine, causing muscle flaccid paralysis. 

BoNTs are defined as Tier 1 toxins by the Federal Select Agent Program due to their extreme lethality [1]. Currently, the only available countermeasure and treatment to botulism is the equine-derived heptavalent botulinum antitoxin (BAT^®^) [20]. As these are foreign proteins, hypersensitivity reactions have been reported, including serum sickness and asystole [20]. In addition, the absence of the Fc fragment in the F(ab)^2^ polyclonal antibodies significantly reduces the half-life in blood to 7.51 h to 34.20 h, depending on the serotype, with the potential for relapses of botulism after treatment [21,22]. In 2003, the FDA approved human botulinum immune globulin (BabyBIG), but because of its limited availability, it is only indicated for treating infant botulism [23]. 

In contrast, we have developed safe and effective monoclonal antibody (mAb) based countermeasures for BoNT/A, B, C, D, and E, which have all completed Phase 1 clinical trials with no drug-related serious adverse effects [24,25,26,27] and have developed a recombinant mAb-based antitoxin for BoNT/F which is in preclinical development [28]. In the current work, we report the identification of mAbs against BoNT/G that, when combined, potently neutralize BoNT/G and thus could be used in the diagnosis, prevention, and treatment of BoNT/G-induced botulism. Based on previous experience showing that three IgGs binding non-overlapping epitopes on BoNT were required to provide protection from intoxication, we set out to identify a potent oligoclonal antibody based BoNT G antitoxin.

## 2. Results

### 2.1. BoNT/G Holotoxin and Domains for Immunization and mAb Characterization

For immunization and mAb characterization, we used commercially available recombinant BoNT/G; full-length catalytically inactive recombinant BoNT/G (BoNT/Gi); recombinant BoNT/G LC-H_N_, LC; and H_C_ domains produced in *E. coli* and then purified. Commercially available BoNT/G holotoxin isolated from *C. argentinense* was also used for immunization.

Hexahistidine-tagged-BoNT/G LC-H_N_ (residues 1–849) was prepared for mouse immunization, and c-Myc-hexahistidine-tagged LC-H_N_ was prepared for library sorting. Both LC-H_N_ domains showed high expression and purity, yielding 5–10 mg/L of culture in the supernatant (Figure S1). BoNT/G LC (residues 1–443) showed high expression and purity (>10 mg/L of culture in the supernatant) (Figure S1). The native BoNT/G H_C_, however, was not well expressed in *E. coli*, so BoNT/G H_C_ fused to a maltose binding protein (MBP) tag was evaluated instead. MBP-fusions to BoNT/G H_C_ residues 873–1297 or 877–1297 were expressed with high yield and purity (>5 mg/L culture; Figure S1).

### 2.2. Mouse Immunization

Three different immunization strategies were used to generate murine mAb-binding BoNT/G, as summarized in Table 1. For the first library, mice were immunized with the recombinant BoNT/G LC-H_N_ domains, followed by boosting with BoNT/G holotoxin produced in *C. argentinense*. For the second library, recombinant BoNT/G LC-H_N_ domains were used for both the initial immunization and for boosting. For the third library, full-length catalytically inactive recombinant BoNT/Gi was used for both the initial immunization and boosting. Blood was harvested before the first immunization and after the third immunization, and ELISA was used to evaluate the immune reaction. The immune serum bound recombinant BoNT/Gi at dilutions up to 10,000 fold (Figure S2). Mouse spleens were subsequently harvested for library construction.

### 2.3. Primary Immune Library Construction and mAb Isolation

Total RNA was isolated from the spleens of immunized mice, and the immunoglobulin heavy (VH) and kappa light chain (Vk) genes were amplified using RT-PCR. The VH and Vk gene repertoires were sequentially cloned into the yeast display vector pYD4, and the ligation mixtures were used to transform *Saccharomyces cerevisiae* EBY100 to create yeast displayed scFv libraries as described previously [29,30]. The library sizes ranged from 2.0–5.0 × 10^7^ (Table 1), and the diversity of the library was confirmed by DNA sequencing of the VH and Vk genes of randomly selected colonies. BoNT/G binding scFv were isolated by sequentially sorting the yeast displayed libraries for four rounds after staining with BoNT/G LC-H_N_ or BoNT/Gi. After four rounds of sorting, individual colonies were sequenced to identify their unique scFv, and the K_D_ of the scFv on the yeast surface was determined by flow cytometry [3,29,31,32]. Sixteen scFv were isolated from the libraries, 13 from the BoNT/Gi library, two from the BoNT/G LC-H_N_ library (6G7 and 6G8), and one from the BoNT/G library (6G6). The K_D_ of the 16 scFv for BoNT/Gi ranged from 3.86 to 102.5 nM with a median K_D_ value of 20.9 nM (Table 2).

### 2.4. BoNT/G Domain Binding and Binding of BoNT/G from C. argentinense 

**Figure 1:**
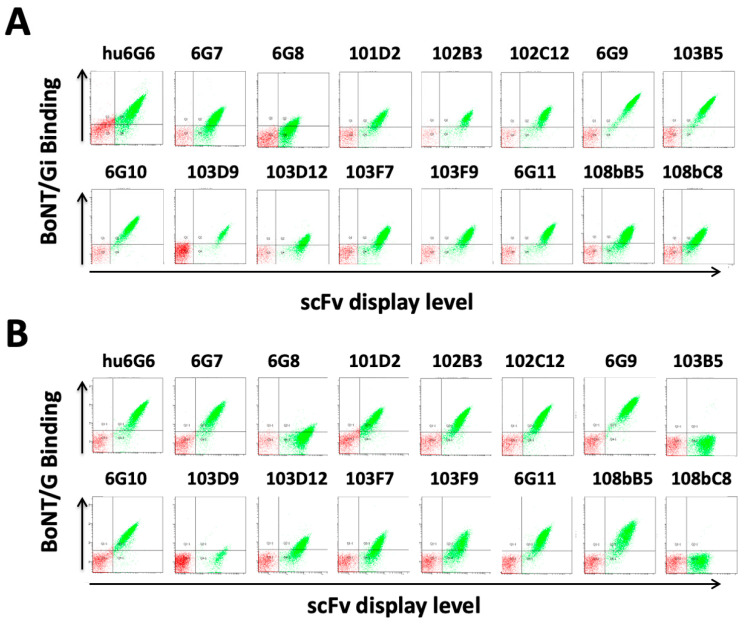
**Binding of scFv to BoNT/Gi and native BoNT/G from *C. argentinense*.** FACS analysis of yeast displayed scFv binding (**A**) BoNT/Gi or (**B**) native BoNT/G. Yeast displayed scFv were stained with (**A**) 50 nM inactive BoNT/Gi, or (**B**) Approximately 50 nM of BoNT/G (based on purity estimated from SDS-PAGE) with binding detected using anti-BoNT/Gi mouse serum. BoNT/Gi or BoNT/G binding mean fluorescence intensity is shown on the *y*-axis, and the mean scFv display fluorescence intensity level is shown on the *x*-axis. A humanized version of 6G6 (hu6G6) was used instead of 6G6 for this experiment.

To confirm that scFv binding BoNT/Gi also bound native BoNT/G, the sixteen yeast displayed scFv were stained with isolated BoNT/G from *C. argentinense* and the binding signal compared to binding to BoNT/Gi. Fourteen of the 16 BoNT/Gi binding scFv (Figure 1A) also bound native BoNT/G Figure 1B), with binding signals consistent with the binding signals after staining with BoNT/Gi. It was not possible to determine a K_D_ value of the scFv for native BoNT/G, as the commercially available BoNT/G was highly impure (see methods section and Figure S3). scFvs 103B5 and 108bC8 bound BoNT/Gi with high affinity (K_D_ value on yeast surface 5.2 nM and 24.0 nM, Figure 1A) but did not show any binding to BoNT/G suggesting they bound a unique epitope on BoNT/Gi that did not exist on the BoNT/G holotoxin.

**Figure 2:**
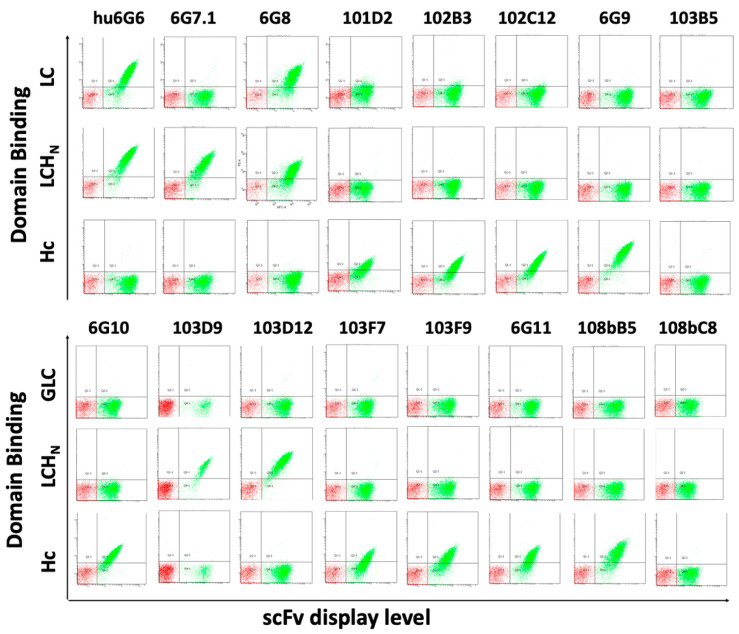
**The BoNT/G domain bound by scFvs.** Yeast displayed scFvs were incubated with 100 nM BoNT/G LC, BoNT/G LC-H_N_ or BoNT/G Hc-MBP, and binding was detected using anti-BoNT/Gi mouse serum and anti-mouse PE. ScFv display level on yeast was quantitated by co-staining with Alexa-647 conjugated anti-SV5 IgG (1:1000). The *x*-axis for each dot-plot indicates increasing scFv display level (mean fluorescence intensity), and the *y*-axis for each plot indicates increasing BoNT/G domain binding (mean fluorescence intensity). A humanized version of 6G6 (hu6G6) was used for this experiment.

Recombinant BoNT/G LC, BoNT/G LC-H_N,_ and BoNT/G Hc-MBP were used to identify the BoNT domain bound by each of the 16 antibodies. Yeast displayed scFvs were incubated with 100 nM of BoNT/G fragments, respectively, followed by detection with anti-BoNT/Gi mouse serum (Figure 2). Two antibodies 6G6, 6G8 bound BoNT/G LC. Three antibodies, 6G7, 103D9, and 103D12, bound BoNT/G LC-H_N_ but not BoNT/G LC, from which we deduced that these three antibodies bound BoNT/G H_N_. Nine antibodies, 6G9, 6G10, 101D2, 102B3, 102C12, 103F7, 103F9, 6G11 and 108Bb5 bound BoNT/G Hc. Finally, the two antibodies 103B5 and 108bC8 that bound BoNT/Gi but did not bind native BoNT/G did not bind any of the three domains (Figure 2). 

### 2.5. Antibody Humanization and Affinity Maturation

To allow for therapeutic use, five lead antibodies (6G6, 6G7, 6G9, 6G10, and 6G11) were humanized and had their affinity for BoNT/Gi increased (affinity maturation) using the steps shown in Table S1 and as described in the methods section in detail. Five antibodies were humanized and affinity matured before in vivo studies because the epitope may change during these processes. These five antibodies were selected based on their binding of the three BoNT/G domains and their affinities for BoNT/Gi. Briefly, humanized VH genes were designed and synthesized for each of the five antibodies and cloned into a yeast displayed human Vk light chain library to produce humanization light chain-shuffling libraries. Each library was selected for BoNT/Gi binding through FACS sorting at high stringency to isolate the humanized scFvs with a fully human light chain. The advantage of using a human light chain library compared to designing a single humanized light chain is that the resulting light chain is fully human, and humanization and affinity maturation could potentially be performed simultaneously. After humanization, hu6G10 had a high affinity for BoNT/G as an scFv of 0.52 nM and an IgG K_D_ of 8.36 pM (Table 3). The affinities of hu6G6, Hu6G7, and hu6G9 (23 nM, 18.8 nM, and 4.5 nM) were further increased by creating and selecting random mutagenesis yeast displayed libraries by flow cytometry, leading to the generation of very high affinity hu6G6.2, hu6G9.1 and Hu6G11.2 with IgG K_D_ of 42.2 pM, 27.48 pM and 48 pM respectively (Table 3). For 6G7, the humanization approach did not yield a humanized scFv. Therefore, the affinity of murine 6G7 was increased using random mutagenesis and yeast display as described above, yielding the higher affinity murine 6G7.1 with an scFv K_D_ increasing from 7.7 nM to 0.27 nM. The 6G7.1 was then humanized by replacing non-critical murine VH and Vk framework residues with human amino acids at these positions and keeping the murine framework residues likely to be critical for binding. The resulting humanized 6G7.1 had a significantly lower affinity than the murine 6G7.1, and therefore, the affinity of Hu6G7.1 was increased by using random mutagenesis, as described above. The resulting humanized antibody, Hu6G7.2, had a very high affinity with an scFv K_D_ value of 1.42 nM and an IgG K_D_ value of 51 pM.

Five antibodies against BoNT/G hu6G6.1, hu6G7.2, hu6G9.1, hu6G10, and hu6G11.2 were identified using the methodologies above. Their affinity to the active BoNT/G holotoxin with IgG K_D_ values ranged from 51 pM to 8.36 pM. 

### 2.6. Identification of Antibodies Binding Non-Overlapping Epitopes

**Figure 3:**
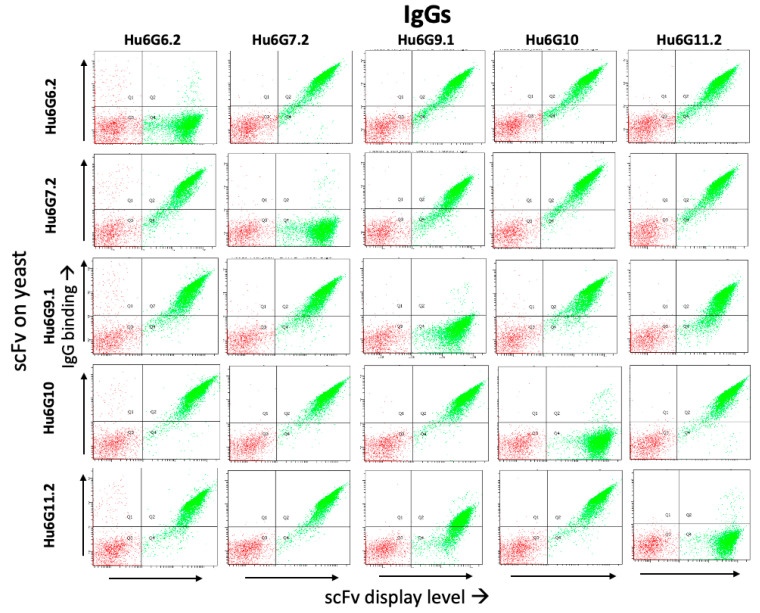
Determination of epitope overlap for the lead antibodies by FACS. Yeast expressing scFv of Hu6G6.2, Hu6G7.2, Hu6G9.1, Hu6G10, and Hu6G11.2 (shown on *y*-axes rows) were incubated with 3 nM BoNT/G, and then with IgG (shown on *x*-axes columns). PE-labeled goat anti-human secondary antibodies and Alexa647-labeled anti-SV5 were used for detection, and the mean fluorescence intensity (MFI) was shown for BoNT/Gi binding (*y*-axis) and scFv display level (*x*-axis).

A flow cytometry sandwich assay [33] using the yeast displayed scFv was used to identify whether the epitopes of hu6G6.1, hu6G7.2, hu6G9.1, hu6G10, and hu6G11.2 overlapped. All five IgG had non-overlapping epitopes, as determined by the FACS competition assays (Figure 3).

### 2.7. In Vivo Mouse Neutralization Assays (MNA)

The mouse neutralization assay (MNA) was employed, rather than in vitro assays, because the mechanism of action of the antibody drugs involves binding and rapid elimination of antibodies from the systemic circulation, which is not recapitulated in a cell-based assay. Given the high impurity (Figure S3) and low specific activity of BoNT/G produced from *C. argentinense* (1.7 × 10^4^ mouse LD_50_s/mg compared to a specific activity of >1.0 × 10^7^ mouse LD_50_s/mg for native BoNT/A), we used recombinant BoNT/G produced from *E. coli* for in vivo mouse neutralization assays. Titration of recombinant BoNT/G in cohorts of 10 mice gave an LD_50_ of 289 pg (95% CI 234–374 pg) or a specific activity of 3.46 × 10^6^ mouse LD_50_s/mg of BoNT/G (Figure S4). In prior studies of the neutralization potency of mAbs to BoNT/A [34], B [35], C, D [33], E [36], and F [28], we observed that single mAbs did not potently neutralize BoNT while combinations of three antibodies binding non-overlapping epitopes potently and stoichiometrically neutralized BoNT. Thus, to minimize the use of animals in these studies, we only determined the end point and ED_50_ for the 10 possible three-IgG combinations. Studies were designed more selectively for single antibodies and mAb pairs to demonstrate the relative potency to three IgG combinations while minimizing animal use.

The mouse neutralization assay was used to evaluate the ability of a 50 μg dose of each IgG to neutralize 200 LD_50_s of BoNT/G (Table 4). The choice of the antibody and recombinant BoNT/G dose was based on prior studies showing that most individual BoNT antibodies did not neutralize BoNT at higher challenge doses [33,34]. The IgG Hu6G6.2 completely protected mice challenged with 200 mouse LD_50_s. However, increasing the challenge dose to 500 mouse LD_50_s resulted in all mice dying. For the other four IgGs, all the mice died at the 200 LD_50_ challenge dose, with the time to death varying significantly, from being slightly longer than the control for hu6G10 to more than 20 times the control for hu6G9.1 (Table 4).

**Figure 4:**
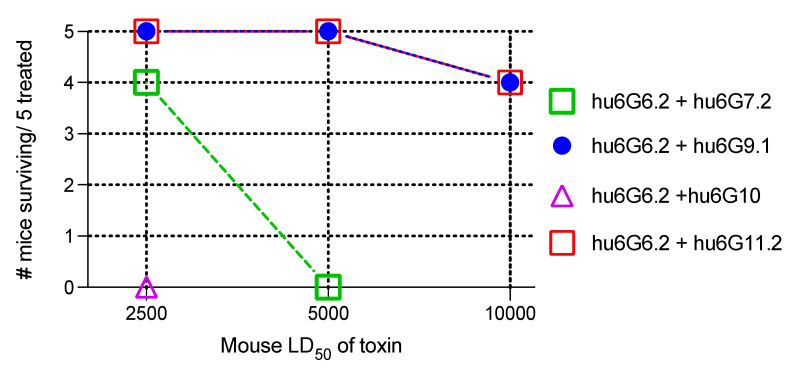
The potency of IgG pairs that included hu6G6.2 (50 μg) in the recombinant BoNT/G neutralisation.

Since hu6G6.2 was the most potent individual mAb, we then studied the potency of the four possible mAb pairs containing hu6G6.2. The initial LD_50_ challenge dose using 50 μg of total mAb was 2500 mouse LD_50_s based on prior studies of the relative potencies of IgG pairs compared to single IgGs. For the antibody pair hu6G6.2 and hu6G10, all the mice died at this challenge dose, while the mice all survived if they received the combination of hu6G6.2 with either hu6G9.1 or hu6G11.2. Four of five mice receiving hu6G6.2 and hu6G7.2 survived (Figure 4). A 25 μg total antibody dose for the IgG pair hu6G6.2 and hu6G11.2 protected two of five mice from a 10,000 mouse LD_50_ challenge, while the same dose of the mAb pair hu6G6.2 and hu6G9.1 protected four of the five mice. Using just 12.5 μg total antibody for the mAb pair hu6G6.2 and hu6G11.2 protected two of five mice from a 10,000 mouse LD_50_ challenge, while the pair hu6G6.2 and hu6G9.1 protected none of the five mice. Note that the relative potencies of the mAb pairs are consistent with the relative prolongation of the time to death of the non-hu6G6.2 mAb in the pair.

**Figure 5:**
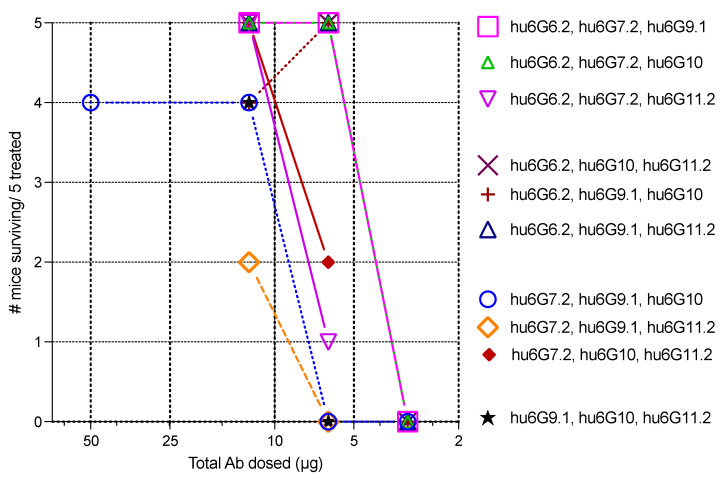
The potency of ten possible three-antibody combinations against challenge with 10,000 LD_50_ of recombinant BoNT/G.

Previous studies indicated that three IgG with high affinity and non-overlapping epitopes significantly improved the potency of in vivo BoNT neutralization over single antibodies or pairs of antibodies [28,33,34,36]. All three-mAb combinations of Hu6G6.2, Hu6G7.2, hu6G9.1, hu6G10, and hu6G11.2 were tested in mice exposed to 10,000 MLD_50_s of recombinant BoNT/G toxin. As shown in Figure 5, the three-antibody combinations of the five antibodies protected mice completely from a 10,000 MLD_50_ challenge of recombinant BoNT/G at a total IgG dose of 12.5 μg/mouse. When the dose of antibodies was reduced to 6.25 μg/mouse, five combinations of the three-antibodies completely protected mice challenged with 10,000 MLD_50_s (hu6G6.2, hu6G7.2, hu6G9.1; hu6G6.2, hu6G7.2, hu6G10; hu6G6.2, hu6G9.1, hu6G11.2; hu6G6.2, hu6G10, hu6G11.2; and hu6G7.2, hu6G10, hu6G11.2. At a dose of 3.125 μg, none of the different combinations were protective, and all the mice died. Thus, the ED_50_ for these three-IgG combinations is approximately 4.69 μg of IgG to neutralize 10,000 LD_50_s.

## 3. Discussion

Mouse immunization followed by construction and sorting of yeast displayed scFv antibody libraries yielded 14 antibodies binding native BoNT/G. At least one antibody bound each of the three BoNT/G domains with affinities typical of the secondary immune response. Two antibodies bound recombinant BoNT/Gi but not native BoNT/G or any of the recombinant domains, suggesting slight differences between the structures of native BoNT/G and BoNT/Gi and the BoNT/G domains. Mutations made to eliminate catalytic activity in BoNT/Gi were at the active site: E231A, R369A, and Y372F. Structural differences between holotoxin and recombinant domains have been observed for BoNT/A. Antibodies were identified that bound recombinant BoNT/A LC but did not bind the native BoNT/A holotoxin [30].

Immunization with the recombinant BoNT/G LC-H_N_ domain alone yielded only two antibodies, while boosting with native BoNT/G holotoxin yielded one antibody. It is not possible to use native BoNT/G as the initial immunogen, as it will kill the mice at sub-immunogenic doses. To avoid killing mice during the immunization process, a protective immune response must first be generated. A toxoid of the BoNT/G can be used, producing a toxoid that can destroy epitopes. In contrast, using domain fragments or catalytically inactive recombinant toxin (BoNT/Gi) avoids killing the mice during immunization. The latter approach allowed the generation of 11 additional antibodies. Of note, nine of the eleven antibodies generated with BoNT/Gi bound the Hc, suggesting that this is the immunodominant domain and potentially explaining why so few mouse antibodies were generated using the BoNT/G LC-H_N_ domain as an immunogen.

To generate therapeutic antibodies from the murine antibodies, five antibodies were humanized and affinity matured. While CDR grafting into a human homology framework is widely used for humanization, we did not use it here for four of the five antibodies. The major disadvantage of CDR grafting is the loss of affinity because of incompatibilities between the mouse CDRs and the human frameworks, requiring back-mutation to the mouse sequence to recover affinity [37]. Instead, four antibodies were humanized using an approach where the murine light chain is replaced by a library of human light chains and binding humanized antibodies isolated by flow cytometry. The advantages of this method include (1) The humanized antibodies include a fully human Vk including the complementarity determining regions and thus significantly fewer murine amino acids; (2) There is simultaneous affinity maturation as evidenced by the affinity of the humanized antibodies is significantly enhanced compared with the parental mouse mAb; (3) The same human Vk library can be used for humanization of any non-human mAb. The disadvantage of this method is that epitope drift can occur [38,39,40].

In vivo, 50 μg of individual IgGs prolonged the time to death with BoNT/G challenge doses of 200 to 500 LD_50_s. Of note, the mAb that bound the BoNT/G LC was significantly more potent than mAb binding the BoNT/G binding domain. It is generally thought that antibodies binding the Hc are more potently neutralizing since Hc antibodies can block BoNT/G uptake by presynaptic neurons. In contrast to single antibodies, a 6.26 μg total dose of three mAb combinations completely protects mice at a 10,000 MLD_50_ challenge dose of BoNT/G, yielding a calculated ED_50_ of 10,000 MLD_50_s/4.69 μg of mAb, which translates to 213 International Units (IU)/mg of antibody where one IU neutralizes 10,000 MLD_50_s. Based on this calculation, a three-mAb dose of 2.8 mg would equate to the 600 IU therapeutic dose of BAT for BoNT/G [20].

We have previously used antibody diversity libraries and display technologies to generate panels of antibodies to BoNT/A [34], B [29], C, D [33], E [36], and F [41] and show that for each of these serotypes, three-mAb combinations result in highly potent BoNT neutralization. Such antibodies can be GMP manufactured and combined as three-mAb combinations. When studied in Phase 1 clinical trials, they showed no serious drug-related adverse events for mAb combinations against serotype A [24], serotype B [25], serotypes C and D [26], or serotype E [27]. In contrast, the current FDA-approved therapy, equine-derived BAT^®^ [20] is immunogenic, and hypersensitivity reactions have been reported, including cardiac arrest and serum sickness.

## 4. Conclusions

Given the prior development of recombinant antitoxins to BoNT/A, B, C, D, E and F, the discovery of a fully human recombinant BoNT/G antitoxin is the final step to enabling the development of a fully recombinant heptavalent anti-BoNT antitoxin and provides an alternative to BAT that is likely to be safer, is renewable, can avoid the cold chain, can be administered both prophylactically and therapeutically and can be administered subcutaneously or intramuscularly and not just intravenously. This potentially allows for the replacement of legacy equine antitoxin in the Strategic National Stockpile. Finally, the individual BoNT/G antibodies can be used in diagnostic assays to detect BoNT/G [42,43].

## 5. Materials and Methods

### 5.1. Ethics

Mouse studies were conducted at the United States Department of Agriculture (USDA) Agricultural Research Service (Albany, CA, USA). Protocols were approved by the USDA Western Regional Research Center Care and Use Committee under protocols 21-8 (Bioassays for the study of botulinum neurotoxins), approved 19 July 2021, and Protocol 20-1 (Production of monoclonal antibodies) approved 27 June 2019. The Albany USDA complies with the guidelines of the Animal Welfare Act and the Public Health Service. The mice used in this research project were housed and treated in strict accordance with the NIH Guide for the Care and Use of Laboratory Animals.

### 5.2. Materials

The Saccharomyces cerevisiae strain EBY100 was used for single-chain variable fragment (scFv) display and scFv library construction. The Escherichia coli (*E. coli*) strain DH5α was used for subcloning and plasmid preparation, BL21 strain, BoNT/G fragment expression, and TG1 strain for soluble scFv preparation. Chinese hamster ovary (CHO) cells were used for immunoglobulin G (IgG) expression.

The yeast peptone dextrose (YPD) medium was used for EBY100 growth, the selective growth dextrose casamino acids media (SD-CAA) for recombinant EBY100 selection, and the selective growth galactose casamino acids media (SG-CAA), for induction of scFv expression. 2×YT media was used for *E. coli* growth.

The holotoxin BoNT/G and toxin complex isolated from *C. argentinense* were purchased from Metabiologics Inc. (Madison, WI, USA). However, we found that the BoNT/G holotoxin labeled as pure was contaminated with other proteins and contained only ~5% BoNT/G (Figure S4). Therefore, we used recombinant BoNT/G, either catalytically active (BoNT/G) or catalytically inactive (BoNT/Gi), purchased from Toxogen GmbH (Hannover, Germany). BoNT/G LC, LC-H_N_, LC-H_N_-myc, and H_C_ fused to maltose binding protein (Hc-MBP) were prepared in BL21. Mouse anti-SV5 antibody was purified from a hybridoma cell line. All the secondary antibodies, including Phycoerythrin (PE) or Allophycocyanin (APC)-conjugated goat anti-human-Fc, goat anti-mouse Fc and goat anti-human F(ab), were purchased from Jackson ImmunoResearch Laboratories (West Grove, PA, USA).

### 5.3. Preparation of BoNT/G fragments and IgGs

The cDNA fragments of BoNT/G, including the BoNT/G light chain (LC, 1-443), N-terminal portion of the light chain (LC-H_N_, 1-849), and C-terminal portion of the heavy chain (H_C_, 865-1297) were synthesized by GeneScript Biotech (Piscataway, NJ, USA). The cDNA fragments were subcloned into the plasmid pET28b with a hexahistidine (His) tag. The fragments were expressed in the *E. coli* strain BL21 with 0.5 mM IPTG induction at 18 °C. The cells were lysed with sonication in lysis buffer (50 mM Tris-Cl, 100 mM NaCl, 1 mM dithiothreitol (DTT), 1 mM EDTA, 5% Glycerol, pH 8.0). BoNT/G recombinant fragments were purified with His-Trap columns on an ÄKTA Avant fast protein liquid chromatography (FPLC) system (GE Healthcare, Pittsburg, PA, USA). The IgGs were generated by subcloning VH and VK genes into a mammalian expression vector with human heavy and kappa light chain constant regions, establishing stable CHO cell lines by transfection and purification of IgG by Protein A chromatography, as previously reported [34].

### 5.4. Mouse Immunization

Female Balb/c mice were immunized with BoNT/G LCH_N_, “pure” native BoNT/G, or catalytically inactive BoNT/G (BoNT/Gi) at three-week intervals three times (Days 0, 21, 42) and boosted once (Day 63). Three groups of four mice each were used for immunization using three immunogens, (1) Immunized and boosted with BoNT/G LCH_N_; (2) Immunized with BoNT/G LCH_N_ and boosted with 1–2 MLD_50_ of Metabiologics BoNT/G; (3) Immunized and boosted with BoNT/Gi. Antibody titers after the third vaccination were evaluated using ELISA. The mice were euthanized, and spleens were removed three to five days after their final immunization and processed to extract mRNA for scFv library construction.

### 5.5. scFv Library Construction and FACS Screening

Three primary immune scFv libraries were constructed for the BoNT/G mAb discovery campaign (Table 1). Briefly, total RNA was isolated from the spleens of immunized mice, from which cDNA was synthesized, and VH and Vk gene repertoires were amplified with a high-fidelity enzyme PicoMaxx (Agilent Technologies Inc., Santa Clara, CA, USA). Library construction was completed as previously described by sequentially cloning VH and Vk genes into the vector pYD4 and transforming yeast cells EBY100 [28]. Transformed EBY100 cells were cultured in SD-CAA at 30 °C for 48 h and then induced in the medium SG-CAA at 18 °C for 48 h with shaking. In parallel, 10 μL of cells were plated on SD-CAA plates and cultured at 30 °C for 48 h to determine the library complexity and size, as previously described [28].

Monoclonal scFv antibodies to BoNT/G were isolated from the primary immune libraries by staining yeast libraries as previously described [28] using 50 nM of BoNT/Gi or BoNT/G LCH_N_ with one hour of incubation at room temperature. Stained yeast cells were washed and incubated with equine anti-BoNT/G serum (1:2000 dilution) for 1 h at 4 °C, washed, and then incubated with 1 μg/mL of Alexa Fluor-647-labeled goat anti-horse Fc antibody (Jackson ImmunoResearch) and 1 μg/mL Alexa Fluor-647-labeled anti-SV5 mAb. All the yeast libraries were sorted with a FACS Aria (BD Biosciences) with the gate set to capture yeast displaying scFv and binding BoNT/Gi or BoNT/G LC-H_N_. After three to four rounds of sorting, individual colonies were picked, grown, and induced for further characterization.

### 5.6. scFv Binding Confirmation and IgG Affinity Determination

To identify unique scFv, the full-length scFv gene was amplified from yeast cells using PCR and sequenced (Elim Bio, Hayward, CA, USA). The K_D_ value of yeast displayed scFv was measured by flow cytometry, as previously described [31]. Briefly, 2 × 10^6^ yeast were incubated in serially diluted BoNT/Gi (50 nM, 10 nM, 2 nM, 0.4 nM, 0.08 nM, 0 nM) for 1 h at room temperature, washed, and then incubated with either mouse anti-BoNT/G polyclonal antibody or one of the humanized BoNT/G IgG, washed again and then incubated with R-phycoerythrin (PE)-conjugated goat anti-human or anti-mouse antibody together with Alexa Fluor^®^ 647-labeled SV5 tag antibody. The mean fluorescence intensity (MFI) was measured by flow cytometry as previously described [29,30]. K_D_ values of IgGs in solution were measured in the solution phase using flow fluorimetry in a KinExA (Sapidyne Instruments, Boise, ID, USA) as described [14,30] using BoNT/Gi or BoNT/G as the antigen.

### 5.7. Epitope Overlap Determination

The overlap of mAb epitopes was determined in a sandwich assay using yeast displayed hu6G6.2, hu6G7.2, hu6G9.1, hu6G10, and hu6G11.2 scFv and their IgGs. Yeast displayed scFv were incubated with 3 nM BoNT/G at RT for 1 h, then washed three times with FACS buffer. Washed yeast cells were aliquoted into wells of a 96-well V-plate. Purified IgGs were added (1 µg/mL, 50 µL/well) into the well. The plate was incubated at 4 °C for 1 h with occasional shaking. Yeast cells were washed with FACS buffer three times before adding PE-labeled goat anti-human IgG secondary Ab and Alexa647-labeled anti-SV5 antibody. Yeast cells were washed three times with FACS buffer and analyzed by FACS using an LSR II cytometer (BD Biosciences, East Rutherford, NJ, USA).

### 5.8. Antibody Humanization and Affinity Maturation

#### 5.8.1. Humanization of Antibodies 6G6, 6G7, 6G9, 6G10 and 6G11

The human germline VH most like the mouse VH gene of 6G6, 6G7, 6G9, 6G10, and 6G11, was identified using IMGT’s [39] Domain Gap Align tool for antibody humanization. The mouse VH was humanized by replacing the framework murine amino acids with those found in the aligned human sequences while retaining the murine complementarity determining regions. Additional murine framework amino acids known to impact complementarity, determining region stability, were also retained where necessary. The humanized VH was then synthesized and co-transformed into EBY 100 using gap repair with a human Vk gene repertoire amplified from BoNT/A, B, C, D, and E toxoid-immunized human donors and cloned into the vector pYD4 to form five VH humanized-human Vk light chain shuffling libraries. For the first round of sorting, each chain shuffled library was grown, induced, and stained with 50 nM BoNT/Gi. Stained yeast cells were washed and incubated with equine anti-BoNT/G serum (1:2000 dilution) for 1 h at 4 °C, washed, and then incubated with 1 μg/mL of Alexa Fluor-647-labeled goat anti-horse Fc antibody (Jackson ImmunoResearch) and 1 μg/mL Alexa Fluor-647-labeled anti-SV5 mAb. All the yeast libraries were sorted with a FACS Aria (BD Biosciences) with the gate set to capture yeast displaying scFv and binding BoNT/Gi or BoNT/G LC-H_N_. After three to four rounds of sorting using decreasing concentrations of antigen, individual colonies were picked, grown, and induced for further characterization by DNA sequencing and measurement of yeast displayed scFv affinity as described above.

#### 5.8.2. Humanization of 6G7.1

The humanization of 6G7 using the chain shuffling approach described above did not yield a humanized antibody. Therefore, the higher affinity 6G7.1 scFv was humanized using traditional CDR grafting. The human germline VH and Vk gene, most like the mouse VH and Vk gene of 6G7.1, was identified using IMGT’s [39] Domain Gap Align tool for antibody humanization. The murine VH and Vk genes were humanized by replacing the framework murine amino acids with those found in the aligned human sequences while retaining the murine complementarity determining regions. Additional murine framework amino acids that impact complementarity, determining region stability, were also retained where necessary. The humanized VH and Vk genes were then synthesized and sequentially cloned into the vector pYD4 to create a humanized 6G7.1 scFv. scFv display was induced, and the affinity for BoNT/Gi was determined as described above.

#### 5.8.3. Affinity Maturation of Antibodies

The scFv of selected antibodies had their affinity for BoNT/G increased by creating yeast displayed libraries of random mutants and selecting for higher affinity binding as previously described [41]. Briefly, scFv genes were amplified with an error-prone PCR with the enzyme Paq 5000 (Agilent), containing 0.05 mM MnCl_2_ in the reaction buffer, and then cloned into pYD4 by gap repairing in EBY100. scFv display was induced as described above, and libraries were incubated with BoNT/Gi at decreasing concentrations starting at 50 nM and decreasing to 0.5 nM in subsequent rounds of staining and sorting. After staining, approximately 0.1% of the BoNT/G binding population was gated, sorted, and grown for the next round of sorting. After three to four rounds of sorting, individual colonies were picked, sequenced, scFv display induced, and the affinity for BoNT/Gi determined by flow cytometry as described above.

### 5.9. In Vivo Mouse Neutralization Assay (MNA) and MLD_50_ Determination

The MNA was performed using groups of 5 mice as described in [44,45]. Briefly, antibody combinations were mixed with the noted number of MLD_50_s of recombinant BoNT/G and injected into female CFW mice (4–5 weeks old) intraperitoneally (IP). The toxin-exposed mice were observed at least twice daily. Most animals receiving lethal doses of BoNT/G become moribund within 12 h, frequently within 4 h without antitoxin. MLD_50_ was determined using groups of 10 mice and calculated with a least-squares regression using Prism for iOS v9.2 (GraphPad Software, LLC, San Diego, CA, USA).

## 6. Patents

The antibodies described here are the subject of patent filings by the Regents of the University of California.

## Figures and Tables

**Table 1:** scFv yeast displayed libraries used for BoNT/G mAb discovery.

Library Name	Immunogen (Days 0, 21, 42)	Boost Immunogen (Day 63)	Library Size	Resulting Antibodies
BoNT/G library	Recombinant BoNT/G LC-HN	2 MLD_50_ BoNT/G holotoxin	2.0 × 10^7^	6G6
BoNT/G LC-HN library	Recombinant BoNT/G LC-HN	Recombinant BoNT/G LC-HN	2.4 × 10^7^	6G7, 6G8
BoNT/Gi library	Recombinant BoNT/Gi	Recombinant BoNT/Gi	5.0 x 10^7^	13 antibodies (see Table 2)

**Table 2:** Characteristics of scFvs binding BoNT/Gi.

Antibody	K_D_ Value for BoNT/Gi (nM)	Qualitative Binding to BoNT/G Holotoxin	BoNT/G Domain Bound
6G6	17.80 ± 3.54	+	LC
6G7	7.72 ± 1.77	+	H_N_
6G8	>100	+	LC
101C2	11.3 ± 0.81	+	Hc
102B3	13.02 ± 19.3	+	Hc
102C12	43.2 ± 3.2	+	Hc
6G9	13.66 ± 1.45	+	Hc
103B5	5.2 ± 0.9	−	−
6G10	3.86 ± 0.6	+	Hc
103D9	44.8 ± 6.5	+	H_N_
103D12	46.3 ± 9.6	+	H_N_
103F7	49.3 ± 8.7	+	Hc
103F9	148 ± 19	+	Hc
6G11	102.5 ± 9.5	+	Hc
108bB5	17.3 ± 7.2	+	Hc
108bC8	24.0 ± 3.1	−	−

**Table 3:** Antibody characteristics for five lead antibodies, including humanized and affinity-matured versions. K_D_ values of scFv were determined using FACS, and K_D_ values of IgG were determined using KinExA.

Antibodies		Domain Bound	K_D_ of scFv on Yeast by FACS for BoNT/Gi	K_D_ of IgG by KinExA for BoNT/G Holotoxin
6G6	6G6	LC	17.8 nM	Not determined
	hu6G6		23.0 nM	8.37 nM
	hu6G6.1		3.4 nM	0.47 nM
	Hu6G6.2		0.22 nM	42.2 pM
6G7	6G7	H_N_	7.7 nM	Not determined
	6G7.1		0.27 nM	26.6 pM
	Hu6G7.1		3.20 nM	1.19 nM
	Hu6G7.2		1.42 nM	51.01 pM
6G9	6G9	Hc	13.66 nM	1.59 nM
	Hu6G9		18.8 nM	Not determined
	Hu6G9.1		2.04 nM	27.48 pM
6G10	6G10	Hc	2.48 nM	0.33 nM
	Hu6G10		0.52 nM	8.36 pM
6G11	6G11	Hc	104.9 nM	Not determined
	hu6G11		4.5 nM	531 pM
	Hu6G11.1		0.32 nM	100.7 pM
	Hu6G11.2		0.56 nM	48 pM

**Table 4:** In vivo neutralization of recombinant BoNT/G by individual IgG (50 μg) against recombinant BoNT/G at 200 MLD_50_ and 500 MLD_50_.

Treatment 50 μg Total Antibodies	Number of Deaths/ 5 Mice Treated 200 MLD_50_	Time to Death (h) 200 MLD_50_	Number of Deaths/ 5 Mice Treated 500 MLD_50_	Time to Death (h) 500 MLD_50_
Control (BoNT/G only)	5/5	3.92 ± 0.52	5/5	3.44 ± 0.28
hu6G6.2	0/5	N/A	5/5	45.9 ± 4.25
hu6G7.2	5/5	19.5 ± 0.00	Not determined	
hu6G9.1	5/5	81.60 ± 9.04	Not determined	
hu6G10	5/5	5.56 ± 0.82	Not determined	
hu6G11.2	5/5	59.4 ± 10.1	Not determined	

## Data Availability

Antibody sequences are not disclosed and will be published in subsequent patent filings. All other data supporting this study are all found within this manuscript and Supplementary Materials.

## Supplementary Materials

The following are available online at https://www.mdpi.com/article/10.3390/toxins15050316/s1, Table S1. Steps in humanization and affinity maturation for the lead mAbs. Figure S1. BoNT /G fragment preparation. Figure S2: ELISA of mouse serum following immunization with BoNT/Gi; Figure S3: Metabiologics BoNT/G holotoxin characterization. Figure S4. Determination of mouse LD_50_ of recombinant BoNT/ G.
