# Supplementary material for: A Three-Monoclonal Antibody Combination Potently Neutralizes BoNT/G Toxin in Mice
Source: Toxins (Basel). 2023 Apr 30;15(5):316. doi: 10.3390/toxins15050316 (PMC10222606; doi:10.3390/toxins15050316)
Supplement: Supplementary file 1 [file toxins-15-00316-s001.zip › toxins-2276291-supplementary.pdf]

toxins

MDPI

A three-monoclonal antibody combination potently neutralizes BoNT/G toxin in mice

Yongfeng Fan, Jianlong Lou, Christina C. Tam, Weihua Wen, Fraser Conrad, Priscila Leal da Silva Alves, Luisa W. Cheng, Consuelo Garcia-Rodriguez, Shauna Farr-Jones and James D. Marks

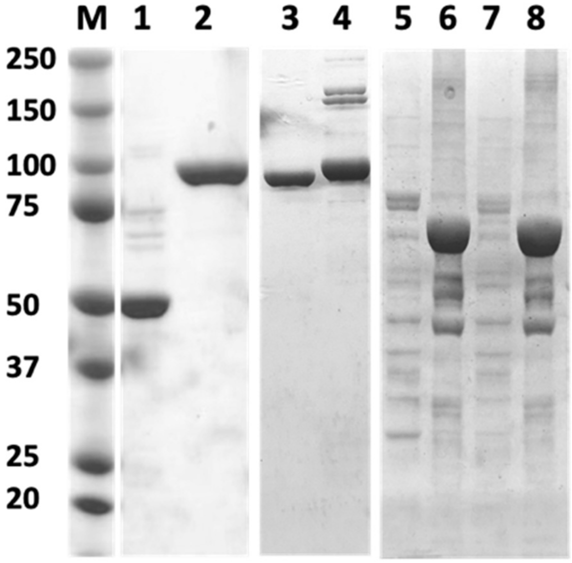

Figure S1. BoNT/G fragment preparation, SDS PAGE analysis.

SDS-PAGE analysis of the preparation of BoNT/G fragments LC, LCH_N and Hc. BoNT/G LC (50kDa) showed high expression yield and good purity (lane 1, LC in reduced buffer showing a monomer with 50kDa; lane 2, LC in non-reduced buffer showing a dimer with 100kDa). G LCH_N (100kDa) also had good expression yield (lane 3, LCH_N and lane 4, LCH_N-myc fusion protein). Hc expression was low. Hc variants with various tags. Lane 5: His-tagged GHc 873-1297. Lane 6: MBP-fused Hc 873-1297. Lane 7: His-tagged Hc 877-1297. Lane 8: His tagged Hc 877-1297.

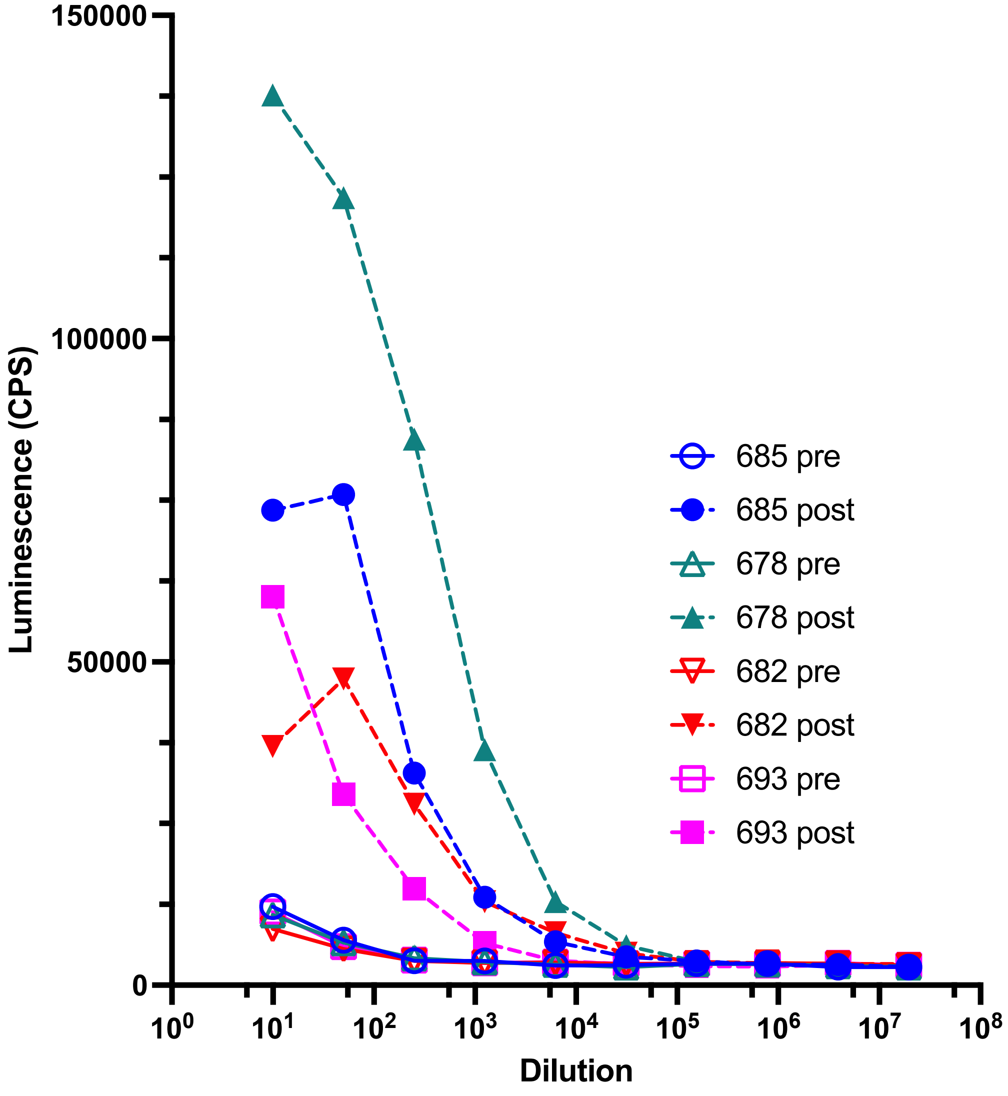

Figure S2. ELISA of mouse serum following mouse immunization with BoNT/Gi.

ELISA plate coated with Toxogen BoNT/Gi. Numbers are mouse identifiers. Open symbols indicate pre-immunization solid symbols, final bleed. The number is the mouse identifier.

Table S1. Steps in humanization and affinity maturation for the lead mAbs.

Starting mAb	Step 1	Resulting mAb	Step 2	Resulting mAbs	Step 3	Resulting mAbs
6G6	Humanization	Hu6G6	Random mutagenesis	Hu6G6.1		
6G7	Random mutagene-sis	6G7.1	humanization	Hu6G7.1	Random mutagenesis	Hu6G7.2
6G9	Humanization	Hu6G9	Random mutagenesis	Hu6G9.1		
6G10	Humanization	Hu6G10	Random mutagenesis			
6G11	Humanization	Hu6G11	Random mutagenesis	Hu6G11.1, hu6G11.2, Hu6G11.3		

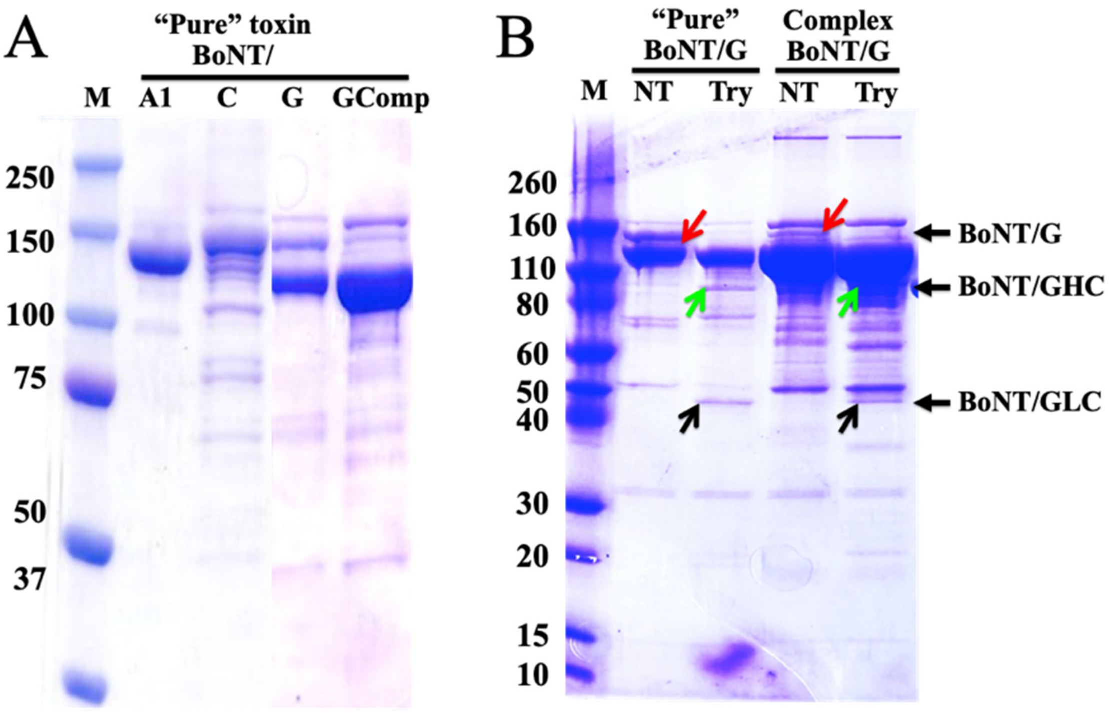

Figure S3. Metabiologics Inc. BoNT/G holotoxin characterization.

Panel A SDS-PAGE of the "pure" BoNT/G and the Complex BoNT/G from Metabiologics Inc. side by side with BoNT/A and BoNT/C. The molecular weight of the major band in BoNT/G is smaller than BoNT/A1 and BoNT/C and similar to BoNT/G complex. **Panel B** shows SDS-PAGE of trypsinized BoNT/G and BoNT/G complex. Compared with non-trypsinized samples, both BoNT/G and BoNT/G complex lost the band at 150 kDa (red arrows) and gained a band at 100kDa (green arrows) and a band at 50 kDa (black arrows). 4 µg of toxin was used for trypsinization at 37°C.

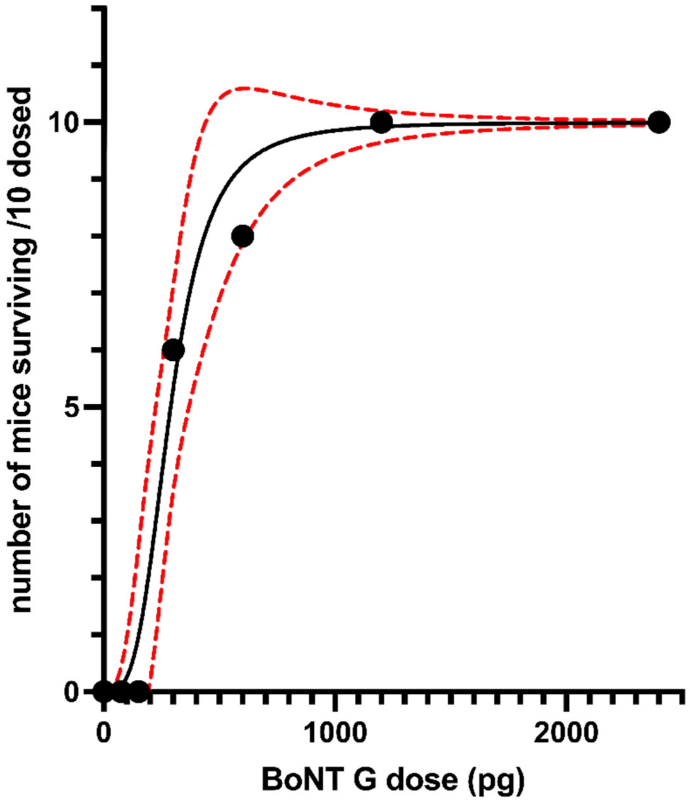

Figure S4. MLD_{50} determination for recombinant BoNT/G (Toxogen GmbH) using least squares regression.

Toxin was delivered intraperitoneally to female mice in cohorts of 10. The dotted lines indicate the 95% confidence interval. $R^2 = 0.978$.

BoNT /G Dose (pg)	Number of mice dead /10 mice dosed
2400	10/10
1200	10/10
600	8/10
300	6/10
150	0/10
75	0/10
0 (Gelatin control)	0/10
